# Long-term Trends in the Prevalence of Congenital Heart Defects in Patients with Down Syndrome in Southern Poland

**DOI:** 10.34763/devperiodmed.20192303.184189

**Published:** 2019-10-27

**Authors:** Artur Dobosz, Mirosław Bik-Multanowski

**Affiliations:** 1 Department of Medical Genetics, Jagiellonian University Medical College, Krakow, Poland

**Keywords:** common atrioventricular canal, trisomy 21, prenatal diagnosis, malformations, wspólny kanał przedsionkowo-komorowy, trisomia 21, diagnostyka prenatalna, malformacje

## Abstract

**Introduction:**

Down syndrome is one of the most common chromosomal abnormalities in humans. Patients have typical dysmorphic features and various congenital malformations. Congenital heart defects were reported as the most common of the latter, occurring in approximately 50% of the cases.

**The aim:**

We aimed to analyse the long-term trends in the prevalence of Down syndrome and related heart defects in the population of southern Poland (Krakow region)

**Material and methods:**

We analysed 500 consecutive patients with Down syndrome who were born from 2006 through 2017 and were diagnosed at the Department of Medical Genetics, Jagiellonian University. Next, we compared our results with the data obtained in previous regional studies.

**Results:**

The prevalence of Down syndrome in the assessed period was 1.65 per 1,000 live births and was similar to the historical prevalence in our region. Cardiac malformations were detected in 57.6% of the patients and the common atrioventricular canal (CAVC) was the most frequent anomaly (35.1%). However, detailed analysis of the frequency of severe heart defects that usually require prompt surgical treatment in the course of infancy revealed that the percentage of CAVC has been significantly lower in recent years (p=0.033).

**Conclusions:**

The prevalence of Down syndrome and the overall frequency of congenital heart defects have not significantly changed in recent years. However, the frequency of CAVC has decreased, which could be related to the technical progress in prenatal detection of this severe anomaly, and to the subsequent elective terminations of affected pregnancies. Further population studies are required to confirm the presence of this trend and elucidate its background.

## Streszczenie

**Wprowadzenie:**

Zespół Downa to jedna z najczęstszych aberracji chromosomowych u ludzi. U pacjentów stwierdza się obecność typowych cech dysmorfii i różnorodnych wad wrodzonych, spośród których w około 50% przypadków występują wrodzone wady serca.

**Cel:**

Celem badania była analiza długoterminowych trendów w częstości występowania zespołu Downai współwystępujących wad serca w populacji południowej Polski (region Krakowa).

**Materiał i metody:**

W badaniu przeanalizowano dane dotyczące 500 pacjentów z zespołem Downa, urodzonych w latach 2006-2017 i zdiagnozowanych w Zakładzie Genetyki Medycznej Uniwersytetu Jagiellońskiego w Krakowie. Wyniki porównano następnie z historycznymi danymi uzyskanymi w ramach badań prowadzonych wcześniej w naszym regionie.

**Wyniki:**

Częstość zespołu Downa w południowej Polsce wynosiła w badanym okresie 1,65 na 1,000 żywych urodzeń i była podobna do częstości obserwowanej wcześniej w naszym regionie. Obecność wrodzonej wady serca stwierdzono u 57,6% pacjentów. Najczęstszą stwierdzaną anomalią był wspólny kanał przedsionkowo-komorowy (35,1%). Szczegółowa analiza towarzyszących wrodzonych wad serca, które zwykle wymagają interwencji kardiochirurgicznej w wieku niemowlęcym wykazała, że odsetek CAVC istotnie zmniejszył się w porównaniu do wcześniejszego okresu (p=0,033).

**Wnioski:**

Częstość występowania zespołu Downa i ogólna częstość wrodzonych wad serca nie zmieniła się istotnie w ciągu ostatnich lat. Zmniejszyła się liczba przypadków CAVC, co może być związane z postępem technicznym w zakresie prenatalnego wykrywania tej ciężkiej anomalii i, w przypadku jej stwierdzenia, z elektywną terminacją ciąży. W celu ostatecznego potwierdzenia obecności i wyjaśnienia przyczyn zaobserwowanego trendu niezbędne jest przeprowadzenie badań populacyjnych.

## Introduction

Down syndrome (trisomy 21) is a genetic disorder resulting from the presence of an additional (third) copy of chromosome 21 [1]. It is one of the most common chromosomal abnormalities in humans. The prevalence of trisomy 21 increases with advanced maternal age [2, 3]. In comparison to women aged 25 years, women over 40 years of age have an increased risk for maternal meiotic I error (odds ratio 5.2) and for maternal meiotic II errors (odds ratio 51.4), which could result in a pregnancy affected with Down syndrome [4]. The meiotic error is of maternal or paternal origin in 91.60% and 8.39% of the cases, respectively [5]. Altered recombination patterns also play a significant role in maternal nondisjunction. The increased risk of nondisjuncton of chromosomes during meiosis can result from no exchange between chromosomes, a single telomeric exchange, or pericentromeric exchange [6, 7, 8]. The great majority of Down syndrome cases (about 95%) is caused by simple trisomy of chromosome 21, whereas 2% is due to mosaicism, 1-3% to translocations and less than 1% to other reasons [9, 10].

The main features of Down syndrome include intellectual disability, facial dysmorphism, and various congenital malformations. Long-term population studies [11] showed that 64% of the patients with trisomy 21 have at least one major anomaly. Congenital heart defects were reported as the most common malformations, occurring in 44% of the cases. Other malformations were far less frequent and included gastrointestinal system anomalies (6%), musculoskeletal abnormalities (5%), urinary system defects (4%), respiratory system abnormalities (2%), eye and central nervous system anomalies (1% and 0.8%, respectively) and other defects (1.7%). The most common congenital heart defect was the common atrioventricular canal (30% of defects), followed by an atrial septal defect (25%), ventricular septal defect (22%), patent ductus arteriosus (5%), tetralogy of Fallot (3%) and other abnormalities (14%).

Data on the prevalence of Down syndrome and congenital heart defects in the Krakow region (southern Poland) were already published several years ago [12, 13]. However, recent progress in the field of ultrasound imaging offers the possibility for detection of small heart defects in the early period of foetal development. The availability of complementary prenatal tests for early detection of chromosomal abnormalities allows quick confirmation of the presence of Down syndrome in the affected foetuses. This, in turn, can result in the termination of some pregnancies. The above factors could significantly influence the prevalence of Down syndrome cases with severe heart defects.

**The aim** of our study was to analyse the long-term trends in the prevalence of Down syndrome and related congenital heart defects in the population of southern Poland.

## Material and methods

We analysed the medical records of 500 consecutive patients with Down syndrome who were diagnosed in the Department of Medical Genetics, Jagiellonian University Medical College in Krakow, in the years 2006-2017. All the patients with Down syndrome were karyotyped in the Department of Medical Genetics and were subsequently followed up in the outpatient clinics of the University Children’s Hospital. The results of clinical examinations and of additional diagnostic tests, including detailed echocardiograms, were available for all the patients.

In order to calculate the mean prevalence of Down syndrome in the general population of southern Poland in the period 2006-2017 we consulted the official database of Statistics in Poland (www.stat.gov.pl) Next, we analysed the medical records of our patients with regard to the presence and type of congenital heart defects and other major malformations, as defined in EUROCAT guidelines for the registration of congenital anomalies (www.eurocat-network.eu) Finally, we compared our findings with historical data on the prevalence of Down syndrome and congenital heart defects in children with Down syndrome in the Krakow region, which were published in 1976 by Konik and Pietrzyk [12] and in 1999 by Malec et al [13]. We used descriptive statistics and Fisher’s exact test for data analysis.

**Table I:** Specific heart defects in the studied group of patients and in the historical cohort described by Malec et al. Tabela I. Specyfika wad serca w badanej grupie pacjentów i w grupie opisanej przez Malec i wsp.

	This study (500 patients) *Badana grupa (500 pacjentów)*	Study by Malec et al. (100 patients) *Grupa opisana przez Malec i wsp. (100 pacjentów)*	Statistical significance of differences between cohorts (Fisher’s exact test) *Istotność statystyczna między dwiema grupami pacjentów (dokładny test Fishera)*
Congenital heart defects usually requiring early surgical treatment Wrodzone wady serca wymagające *zazwyczaj wczesnej interwencji kardiochirugicznej*	215 (74.6%) (*108)	89 (89%)	
Common atrioventricular canal (CAVC) *Wspólny kanał przedsionkowo-komorowy (CAVC)*	101 (35.1%) (*42)	54 (60.6%)	p=0.032 (*p=0.009)
Ventricular septal defect (VSD) *Ubytek w przegrodzie międzykomorowej (VSD)*	78 (27.1%) (*39)	24 (26.9%)	ns
Tetralogy of Fallot (ToF) *Tetralogia Fallota (ToF)*	13 (4.9%) (*6)	8 (8.08%)	ns
Patent ductus arteriosus (PDA) *Przetrwały przewód tętniczy (PDA)*	19 (6.6%) (*8)	3 (3.3%)	ns
Transposition of great arteries (TGA), isolated abnormalities of pulmonary arteries *Przełożenie wielkich pni tętniczych (TGA), izolowane nieprawidłowości tętnic płucnych*	4 (1.4%) (*3)	0 (0)	ns
Other defects (surgical treatment usually in older children or not required) *Inne defekty (wymagające zazwyczaj zabiegów kardiochirurgicznych w późniejszym wieku lub niewymagające zabiegu)*	73 (25.3%) (*37)	11 (11%)	
Atrial septal defect ostium primum (ASD I) *Ubytek w przegrodzie międzyprzedsionkowej - ostium primum (ASD I)*	10 (13.6%) (*5)	8 (8.08%) (only surgically repaired cases) (tylko pacjenci operowani)	Not assessed Nieoceniane
Atrial septal defectostium secundum (ASD II ) *Ubytek w przegrodzie międzyprzedsionkowej - ostium secundum (ASD II)*	63 (86.2%) (*32)	3 (3.3%) (only surgically repaired cases) (tylko pacjenci operowani)	Not assessed Nieoceniane
No congenital heart defect *Bez wrodzonych wad serca*	212 (42.4%) (*105)	No data Brak danych	

* 250 youngest patients, who were diagnosed from 2012 through 2017; ns – nonsignificant * 250 najmłodszych pacjentów, którzy byli diagnozowani od 2012 do 2017 roku; ns – nieistotne statystycznie

## Results

The mean prevalence of Down syndrome in the Krakow region between 2006 and 2017 equalled 1.65 per 1,000 live births (1 in 605 newborns).

The assessed group consisted of 258 boys and 242 girls. Most of the patients had classic trisomy 21 (94.8%; 474 cases). Robertsonian translocation was detected in 2.8% of the cases (der21;21, der14;21 and der21;22 in 14, 7 and 1 patient, respectively), whereas mosaicism of the 21^st^ chromosome was observed in 2.2% of the cases (11 patients). In one patient, isochromosome 21 was diagnosed.

Cardiac anomalies were detected in 288 patients (57.6% of the cases). Common atrioventricular canal (CAVC) was the most frequent congenital heart defect, and it occurred in 35.1% of the patients. Ventricular septal defect (VSD) was observed in 27.1% of the patients, whereas atrial septal defect was detected in 25.3%. In 20 children, VSD coexisted with ASD. Patent ductus arteriosus (PDA), tetralogy of Fallot (ToF) and other defects accounted for 6.6%, 4.9% and 1.4% of the anomalies, respectively.

Detailed analysis of the frequency of severe heart defects that usually require prompt surgical treatment during infancy (we excluded ASD, which is often treated in older children) revealed that the percentage of CAVC has been significantly lower in recent years, when compared to data published by Malec et al. (60.7% and 47%, respectively; p=0.033). The decrease in the frequency of CAVC was apparent among the youngest patients diagnosed in our centre: whereas in the subgroup of 250 older children (diagnosed from 2006 through 2012) it equalled 51.8%, in 250 younger children (diagnosis from 2012 through 2017) it reached only 41.6% (p=0.009 for the comparison with the cohort described by Malec et al). The frequencies of other severe heart defects did not significantly change since 1999.

We did not compare the historical and current frequencies of ASD, because the historical data related only to some, most severe cases, which required immediate surgery.

**Fig. 1:**
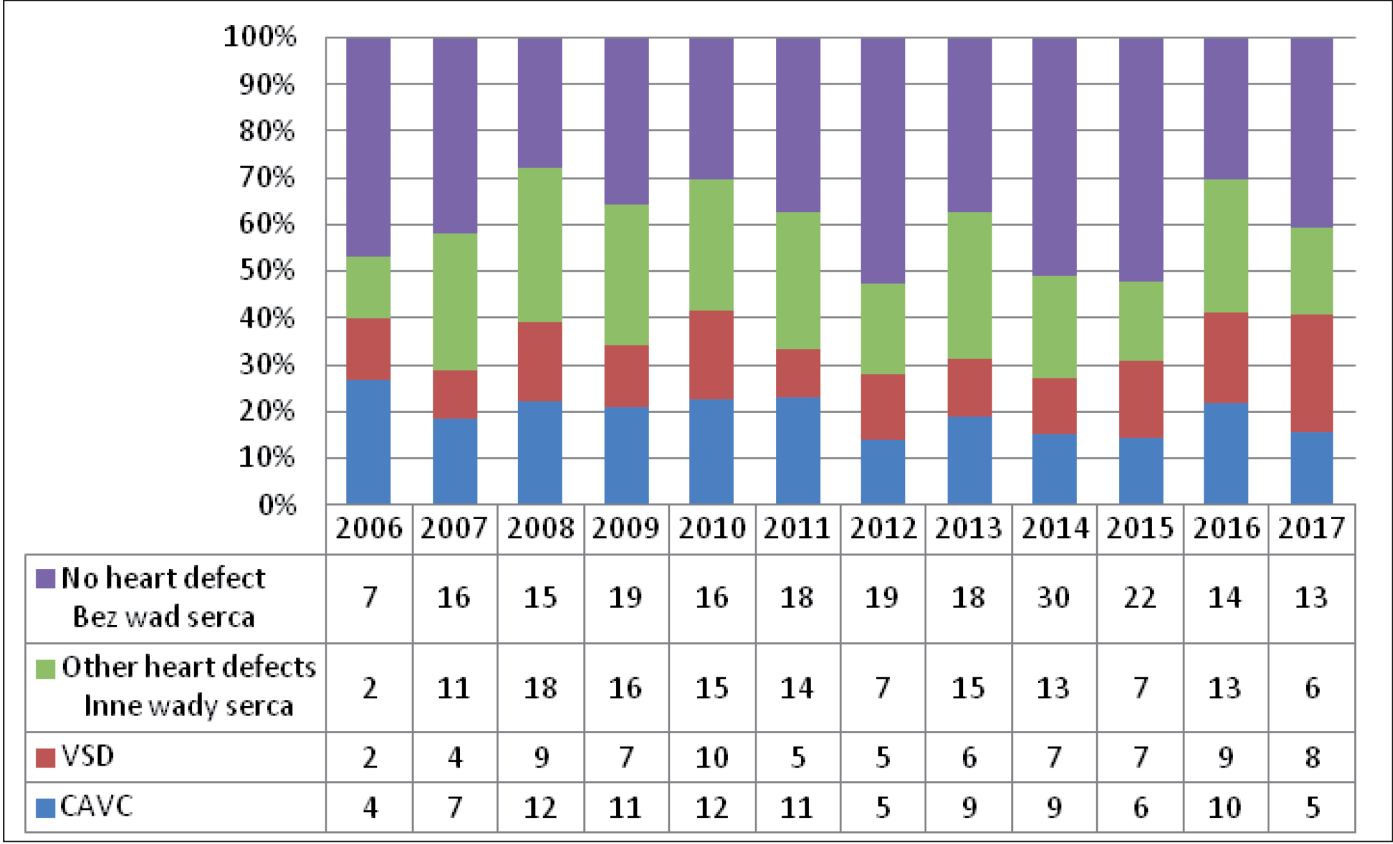
The group of 500 consecutive patients with Down syndrome diagnosed in the period 2006-2017. Ryc. 1. Grupa 500 kolejnych pacjentów z zespołem Downa zdiagnozowanych w latach 2006-2017.

Details on the detected heart defects and the analysed historical data are presented in Table I and in Figure 1.

Additional congenital anomalies in the assessed group of patients included digestive system anomalies (18 cases), urinary system defects (9 cases), eye anomalies (4 cases), central nervous system anomalies (1 case) and haematological findings (28 cases). Other most frequent anomalies, both in the children with and without heart defects, affected hematology, and secondly the digestive system. Details are available in Table II.

**Table II:** Additional anomalies detected in the assessed group of patients. Tabela II. Dodatkowe anomalie stwierdzone u pacjentów w badanej grupie.

	With heart defect *Z wadą serca*	Without heart defect *Bez wady serca*
Gastrointestinal system anomalies *Anomalie układu pokarmowego*		
Hirschsprung *Choroba Hirschsprunga*disease	4	4
Duodenal *Atrezja dwunastnicy* atresia	2	6
Hirschsprung and Duodenal atresia *Choroba Hirschsprunga i atrezja dwunastnicy*	1	0
Anal *Atrezja* atresia *odbytu*	1	0
Total/*Ogółem*	8 2.7%	10 4.8%
Urinary system anomalies *Anomalie układu moczowo-płciowego*		
Cryptorchidism *Wnętrostwo*	3	2
Megaureter *Moczowód olbrzymi*	0	2
Kidney *Hipoplazja* hypoplasia *nerek*	2	0
Total/*Ogółem*	5 1.7%	4 1.9%
Eye anomalies *Anomalie oczu*		
Cataract *Zaćma*	1	3
Total/*Ogółem*	1 0.3%	3 1.4%
Nervous system anomalies *Anomalie układu nerwowego*		
Agenesis of vermis cerebellar *Agenezja robaka móżdżku*	0	1
Total/*Ogółem*	0	1 0.5%
Haematological anomalies *Anomalie hematologiczne*		
Thrombocytopenia *Małopłytkowość*	6	9
Transient myeloproliferative disorder*Przejściowy zespół mieloproliferacyjny*	2	3
ALL	2	3
AML	3	0
Total/*Ogółem*	13 4.5%	15 7.1%

## Discussion

In our study we assessed the trends in the prevalence of Down syndrome and related congenital heart defects in the population of the Krakow region (Małopolska) over many years. Since 2006, a routine prenatal testing program has been offered by the National Health Fund. The Department of Medical Genetics is the only public health care unit in the Krakow region where prenatal karyotyping, comprehensive genetic counseling and subsequent follow-up for children with Down syndrome is available. Thus, it can be assumed that the cohort described in this study represents the vast majority of the population of the affected children in the entire region.

The prevalence of Down syndrome in our study was similar to that described in 1976 by Konik and Pietrzyk (1.55-1.6 per 1000 live births). Interestingly, according to the data from the EUROCAT database, Down syndrome is slightly more frequent in the Krakow region, than in other parts of Poland (1.17 per 1000 live births on average; www.eurocat-network.eu) Our findings might suggest that despite improving the availability of prenatal tests for early detection of chromosomal abnormalities (such as advanced methods of sonographic examination and assessment of cell-free foetal DNA in pregnant women), the frequency of pregnancy terminations due to Down syndrome in southern Poland remains stable. On the contrary, technological progress in the field of prenatal testing might explain the apparent decrease in the frequency of CAVC in recent years. This is because CAVC is one of the most severe heart defects with early onset of circulatory insufficiency and uncertain prognosis regarding the effects of surgical treatment. Therefore, CAVC diagnosis in early pregnancy might increase the chance of elective abortion.

It is interesting that although the percentage of congenital heart defects in our cohort reached almost 57%, without ASD II it fell to 43.6%, which is very similar to the percentage reported by French authors [11]. Better detectability of asymptomatic ASD II in older children with Down syndrome by means of technically advanced echocardiography can explain this finding.

## Conclusions

The prevalence of Down syndrome and the overall frequency of congenital heart defects have not significantly changed over recent years. However, the frequency of CAVC has decreased, which could be related to the technical progress in prenatal detection of this severe anomaly and to subsequent elective terminations of affected pregnancies. Further population studies are required to confirm the presence of this trend and elucidate its background.

## References

[1] Lejeune J, Gautier M, Turpin R (1959). [Study of somatic chromosomes from 9 mongoloid children]. [Article in French]. CR Hebd Seances Acad Sci.

[2] Coppedè F (2016). Risk factors for Down syndrome. Arch Toxicol.

[3] Ghosh S, Feingold E, Dey SK (2009). Etiology of Down syndrome: Evidence for consistent association among altered meiotic recombination, nondisjunction, and maternal age across populations. Am J Med Genet A.

[4] Yoon PW, Freeman SB, Sherman SL, Taft LF, Gu Y, Pettay D, Flanders WD, Khoury MJ, Hassold TJ (1996). Advanced maternal age and the risk of Down syndrome characterized by the meiotic stage of chromosomal error: a population-based study. Am J Hum Genet.

[5] Ballesta F, Queralt R, Gómez D, Solsona E, Guitart M, Ezquerra M, Moreno J, Oliva R (1999). Parental origin and meiotic stage of non-disjunction in 139 cases of trisomy 21. Ann Genet.

[6] Warren AC, Chakravarti A, Wong C, Slaugenhaupt SA, Halloran SL, Watkins PC, Metaxotou C, Antonarakis SE (1987). Evidence for reduced recombination on the nondisjoined chromosomes 21 in Down syndrome. Science.

[7] Lamb NE, Feingold E, Savage A, Avramopoulos D, Freeman S, Gu Y, Hallberg A, Hersey J, Karadima G, Pettay D, Saker D, Shen J, Taft L, Mikkelsen M, Petersen MB, Hassold T, Sherman SL (1997). Characterization of susceptible chiasma configurations that increase the risk for maternal nondisjunction of chromosome 21. Hum Mol Genet.

[8] Lamb NE, Yu K, Shaffer J, Feingold E, Sherman SL (2005). Association between maternal age and meiotic recombination for trisomy 21. Am J Hum Genet.

[9] Flores-Ramírez F, Palacios-Guerrero C, García-Delgado C, Morales-Jiménez AB, Arias-Villegas CM, Cervantes A, Morán-Barroso VF (2015). Cytogenetic profile in 1,921 cases of trisomy 21 syndrome. Arch Med Res.

[10] Morris JK, Alberman E, Mutton D, Jacobs P (2012). Cytogenetic and epidemiological findings in Down syndrome: England and Wales 1989-2009. Am J Med Genet A.

[11] Stoll C, Dott B, Alembik Y, Roth MP (2015). Associated congenital anomalies among cases with Down syndrome. Eur J Med Genet.

[12] Konik R, Pietrzyk JJ (1976). [Congenital anomalies of the developmental age. I. Incidence of malformations in newborn infants diagnosed the 1st week of life in the Cracow Region]. [Article in Polish]. Pediatr Pol.

[13] Malec E, Mroczek T, Pajak J, Januszewska K, Zdebska E (1999). Results of surgical treatment of congenital heart defects in children with Down’s syndrome. Pediatr Cardiol.

